# Novel Bi-heterocycles as Potent Inhibitors of Urease and Less Cytotoxic Agents: 3-({5-((2-Amino-1,3-thiazol-4-yl)methyl)-1,3,4-oxadiazol-2-yl}sulfanyl)-*N*-(un/substituted-phenyl)propanamides 

**DOI:** 10.22037/ijpr.2019.13084.11362

**Published:** 2020

**Authors:** Muhammad Athar Abbasi, Muhammad Shahid Ramzan, Aziz ur-Rehman, Sabahat Zahra Siddiqui, Mubashir Hassan, Syed Adnan Ali Shah, Muhammad Ashraf, Muhammad Shahid, Sung-Yum Seo

**Affiliations:** a *Department of Chemistry, Government College University, Lahore-54000, Pakistan.*; b *Institute of Molecular Biology and Biotechnology, The University of Lahore, Pakistan. *; c *Faculty of Pharmacy, Universiti Teknologi MARA, Puncak Alam Campus, 42300 Bandar Puncak Alam, Selangor Darul Ehsan, Malaysia. *; d *Atta-ur-Rahman Institute for Natural Products Discovery (AuRIns), Level 9, FF3, Universiti Teknologi MARA, Puncak Alam Campus, 42300 Bandar Puncak Alam, Selangor Darul Ehsan, Malaysia. *; e *Department of Chemistry, The Islamia University of Bahawalpur, Bahawalpur-63100, Pakistan. *; f *Department of Biochemistry, University of Agriculture, Faisalabad-38040, Pakistan. *; g *Department of Biological Sciences, College of Natural Sciences, Kongju National University, Gongju, 32588, South Korea.*

**Keywords:** Ethyl 2-(2-amino-1, 3-thiazol-4-yl)acetate, 1, 3, 4-Oxadiazole, Propanamides, Urease, Cytotoxicity, Potent inhibitors

## Abstract

The synthesis of a novel series of bi-heterocyclic propanamides, **7a-l**, was accomplished by *S*-substitution of 5-[(2-amino-1,3-thiazol-4-yl)methyl]-1,3,4-oxadiazol-2-thiol (**3**). The synthesis was initiated from ethyl 2-(2-amino-1,3-thiazol-4-yl)acetate (**1**) which was converted to corresponding hydrazide, **2**, by hydrazine hydrate in methanol. The refluxing of hydrazide, **2**, with carbon disulfide in basic medium, resulted in 5-[(2-amino-1,3-thiazol-4-yl)methyl]-1,3,4-oxadiazol-2-thiol (**3**). A series of electrophiles, **6a-l**, was synthesized by stirring un/substituted anilines (**4a-l**) with 3-bromopropanoyl chloride (**5**) in a basic aqueous medium. Finally, the targeted compounds, **7a-l**, were acquired by stirring **3 **with newly synthesized electrophiles, **6a-l**, in DMF using LiH as a base and an activator. The structures of these bi-heterocyclic propanamides were confirmed through spectroscopic techniques, such as IR, ^1^H-NMR, ^13^C-NMR, and EI-MS. These molecules were tested for their urease inhibitory potential, whereby, the whole series exhibited very promising activity against this enzyme. Their cytotoxic behavior was ascertained through hemolysis and it was observed that all these were less cytotoxic agents. The *in-silico* molecular docking analysis of these molecules was also in full agreement with their* in-vitro *enzyme inhibition data.

## Introduction

Heterocyclic compounds have been under investigation for a long time because of their important pharmacological properties (1). Thiazole is one such important heterocyclic system with pronounced pharmacological activities (2). Thiazole is classified under five-membered heterocyclic class of compounds and is found in many natural and synthetic agents. Naturally, thiazole is available in a large number of terrestrial and marine compounds with different pharmacological activities. Thiazole is also present in the vitamin B1 (Thiamine). In synthetic substituted thiazole derivatives, 2-aminothiazoles have shown a variety of biological activities such as anti-bacterial, antifungal, antitubercular, anti-HIV, anti-inflammatory, anticancer, anticonvulsant, antidiabetic, antihypertensive, antiprotozoal, dopaminergic, plasminogen activator inhi-bitor-1, neuroprotective, and antioxidant. This broad spectrum of activities makes 2-aminothiazole as an attractive moiety in medicinal chemistry (3, 4).

1,3,4-Oxadiazole is another important heterocycle and its different derivatives possess an extensive spectrum of pharmacological activities such as antiviral, antibacterial, antitumor, antituberculosis, anti-inﬂammatory, anticonvulsant, and anti-Alzheimer activities (5−11). There have been much advancement regarding synthesis and investigation of biological activities of 1,3,4-oxadiazole der-ivatives in the last two decades. Several methods have been reported for the synthesis of 1,3,4-oxadiazoles such as reaction of acyl hydrazines with isothiocyanates, reaction of acid hydrazides with carbon disulﬁde in basic medium, cyclodehydration reaction of diacylhydrazines, reaction of hydrazides with orthoesters, cyclization oxidative reaction of *N*-acyl hydrazones, and reaction of *N*-acylbenzotriazoles with acyl hydrazides (12−19). 

Urease is known to be involved in different pathogenic processes. It has been known to be involved in pyelonephritis, peptic ulceration, kidney stone, hepatic encephalopathy, uroli-thiasis, and urinary catheter incrustation (20, 21). The molecular docking analysis approximates the ligands regarding their orientation and conformation at binding site of target protein. The precise forecast of activity and precise structural modeling can be achieved by the docking studies (22). 

One of the key objectives of organic and medicinal chemists is to design and synthesize the molecules having potent therapeutic values. The rapid development of resistance to existing drugs generates a serious challenge to the scientific community. Consequently, there is a vital need for the development of new drugs having potent activity. The rationale in the present study was that minor modification in the structure of such heterocycles can lead to quantitative as well as qualitative changes in their biological activity. So, in continuation of our previous effort to explore the enzyme inhibitory activity of related bi-heterocyclic bi-amides (23), hereby, we report the synthesis of some novel bi-heterocyclic propanamides as potent urease inhibitors with mild cytotoxicity. 

## Experimental

*Chemistry*

All the chemicals, along with analytical grade solvents, were purchased from Sigma-Aldrich, Alfa Aesar (Germany), or Merck through local suppliers. Pre-coated silica gel Al-plates were used for TLC with ethyl acetate and *n*-hexane as solvent system (25:75). The spots were detected by UV_254_. Gallonkamp apparatus was used to detect melting points (uncorrected) in capillary tubes. IR spectra (ν, cm^–1^) were recorded by KBr pellet method in the Jasco-320-A spectrophotometer. EI-MS spectra were measured on a JEOL JMS-600H instrument with data processing system. ^1^H-NMR spectra (δ, ppm) were recorded at 600 MHz (^13^C-NMR spectra, at 150 MHz) in DMSO-*d*_6_ using the Bruker Advance III 600 As- cend spectrometer using BBO probe. The coupling constant (*J*) is given in Hz and chemical shift () in ppm. The abbreviations used in interpretation of ^1^H NMR spectra are as follows: s, singlet; d, doublet; dd, doublet of doublets; t, triplet; br.t, broad triplet; q, quartet; quint, quintet; sex, sextet; sep, septet; m, multiplet; dist., distorted. 

*Procedure for the synthesis of 5-((2-amino-1,3-thiazol-4-yl)acetohydrazide (* ***2*** *)*

Ethyl 2-(2-amino-1,3-thiazol-4-yl)acetate (**1**; 10 g, 0.054 mol) and methanol (200 mL) were taken in a 500 mL RB flask. Hydrazine hydrate (2.5 mL, 0.054 mol) was added drop wise and the mixture was refluxed for 2 h. The reaction progress was observed by TLC using *n*-hexane and ethyl acetate solvent system (40:60). After completion, the reaction mixture was allowed to cool at room temperature to attain white colored precipitates, which were filtered and washed with methanol to obtain purified hydrazide, **2**. 

*Synthesis of 5-[(2-amino-1,3-thiazol-4-yl)methyl]-1,3,4-oxadiazol-2-thiol (* ***3*** *)*

5-((2-Amino-1,3-thiazol-4-yl)acetohydrazide (**2**; 4 g, 0.023 mol) was dissolved in C_2_H_5_OH (70 mL) in a 250 mL RB flask at 28 °C and then solid KOH (1.34 g, 0.023 mol) was dissolved on reflux. Carbon disulphide (3.50 mL, 0.046 mol) was poured drop wise at 28 °C and then the reaction mixture was refluxed again for 5 h. Reaction progress was noted with TLC using *n*-hexane and ethyl acetate solvent system (7:3). After completion of reaction, excess of ethanol was evaporated and sufficient ice cold distilled water was added followed by addition of dilute HCl to adjust pH of 4-5. Light peach colored precipitates of 5-[(2-amino-1,3-thiazol-4-yl)methyl]-1,3,4-oxadiazol-2-thiol (**3**) were filtered and washed with distilled water. 

*Synthesis of 3-bromo-N-(un/substituted-phenyl)propanamides (* ***6a-l*** *)*

The un/substituted anilines (**4a-l**; 0.038 mol) were suspended in 30 mL distilled water in an iodine flask (100 mL) and aqueous Na_2_CO_3_ solution (10%, 2-3 mL) was added. 3-Bromopropanoyl chloride (**5**; 0.038 mol) was added gradually with vigorous manual shaking. Then this mixture was set to stir on magnetic stirrer for 2-3 h. Reaction completion was monitored by TLC. On completion, the excess ice-cold distilled water (60 mL) was added and the resulting precipitates were collected through filtration, washed with distilled water, and dried to get purified electrophiles, **6a-l**.

*General procedure for the synthesis of 3-({5-[(2-amino-1,3-thiazol-4-yl)methyl]-1,3,4-oxadiazol-2-yl}sulfanyl)-N-(un/substituted-phenyl)propanamides *(***7a-l***) 

5-[(2-Amino-1,3-thiazol-4-yl)methyl]-1,3,4-oxadiazol-2-thiol (**3**; 0.1 g, 0.467 mmol) was dissolved in *N,N*-dimethyl formamide (DMF, 5-10 mL) in a 100 mL RB flask. Solid LiH (0.005 g) was added and the mixture was stirred for half an hour. Then, different aforementioned electrophiles, 3-bromo-*N*-(un/substituted-phenyl)propanamides (**6a-l**; 0.467 mmol), were added and the mixture was set to stirring for 3-5 h. The progress of reaction was monitored through TLC using *n*-hexane and ethyl acetate solvent system (80:20). On completion, excess ice-cold distilled water was added and the precipitates obtained were filtered, washed with distilled water, and dried to acquire purified products, **7a-l**.

*3-({5-[(2-Amino-1,3-thiazol-4-yl)methyl]-1,3,4-oxadiazol-2-yl}sulfanyl)-N-phenylpropanamide (* ***7a*** *)*

Light brown solid; Yield: 89%; m.p.:190-191 °C; Mol. Formula: C_15_H_15_N_5_O_2_S_2_; Mol. Mass: 361 gmol^-1^; IR (KBr, *ʋ*/cm^-1^): 3355 (N-H stretching), 3050 (C-H of aromatic ring), 2923 (-CH_2_- stretching), 1665 (C=O stretching), 1645 (C=N stretching), 1577 (C=C stretching of aromatic ring),; ^1^H-NMR (DMSO-d_6_, 600 MHz, *δ*/ppm): 10.06 (s, 1H, -NH-CO-1’’), 7.59 (br.d, 2H, *J* = 7.9 Hz, H-2’’’ and H-6’’’), 7.30 (br.t, *J* = 7.7 Hz, 2H, H-3’’’ and H-5’’’), 7.04 (br.t, 1H, *J* = 7.4, H-4’’’), 7.01 (s, 2H, -NH_2_), 6.42 (s, 1H, H-5), 4.05 (br.s, 2H, CH_2_-6), 3.48 (br.t, 2H, *J* = 6.7, CH_2_-3’’), 2.89 (br.t, 2H, *J* = 6.7 Hz, CH_2_-2’’); ^13^C-NMR (DMSO-d_6_, 150 MHz, *δ*/ppm): 168.73 (C-1’’), 168.68 (C-5’), 165.66 (C-2’), 163.40 (C-2), 143.94 (C-4), 138.38 (C-1’’’), 128.68 (C-3’’’ & 5’’’), 123.22 (C-4’’’), 119.05 (C-2’’’ & C-6’’’), 103.17 (C-5), 35.66 (C-6), 27.68 (C-2’’), 27.52 (C-3’’); EI-MS: m/z 361 [M]^+^, 269 (C_9_H_9_N_2_O_2_S_2_)^+^, 241 (C_8_H_9_N_4_OS_2_)^+^, 214 (C_6_H_5_N_4_OS­_2_)^+^, 113 (C_4_H_5_N_2_S)^+^, 93 (C_6_H_7_N)^+^, 77 (C_6_H_5_)^+^, 55 (C_3_H_3_O)^+^.

*3-({5-[(2-Amino-1,3-thiazol-4-yl)methyl)-1,3,4-oxadiazol-2-yl}sulfanyl)-N-(2-methylphenyl)propanamide (* ***7b*** *)*

Light brown solid; Yield: 85%; m.p.: 201-202 °C; Mol. Formula: C_18_H_17_N_5_O_2_S_2_; Mol. Mass: 375 gmol^-1^; IR (KBr, *ʋ*/cm^-1^): 3360 (N-H stretching), 3045 (C-H of aromatic ring), 2935 (-CH_2_- stretching), 1661 (C=O stretching), 1647 (C=N stretching), 1576 (C=C stretching of aromatic ring); ^1^H-NMR (DMSO-d_6_, 600 MHz, *δ*/ppm): 9.42 (s, 1H, -NH-CO-1’’), 7.37 (br.d, *J *= 7.8 Hz, 1H, H-6’’’), 7.20 (br.d, *J* = 7.3 Hz, 1H, H-3’’’), 7.15 (br.t, *J *= 7.6 Hz, 1H, H-5’’’), 7.08 (br.t, *J* = 7.3 Hz, 1H, H-4’’’), 7.02 (br.s, 2H, -NH_2_), 6.42 (s, 1H, H-5), 4.05 (br.s, 2H, CH_2_-6), 3.47 (br.t, *J* = 6.7 Hz, 2H, CH_2_-3’’), 2.90 (br.t, *J* = 6.7 Hz, 2H, CH_2_-2’’), 2.17 (br.s, 3H, 2’’’-CH_3_); ^13^C-NMR (DMSO-d_6_, 150 MHz, *δ*/ppm): 168.65 (C-1’’), 168.58 (C-5’), 165.55 (C-2’), 163.34 (C-2), 143.71 (C-4), 135.97 (C-1’’’), 131.62 (C-2’’’), 130.16 (C-6’’’), 125.79 (C-5’’’), 125.13 (C-3’’’), 124.94 (C-4’’’), 103.11 (C-5), 35.66 (C-6), 27.86 (C-2’’), 27.40 (C-3’’), 17.75 (2’’’-CH_3_); EI-MS: m/z 375 [M]^+^, 269 (C_9_H_9_N_4_O_2_S_2_)^+^, 241 (C_8_H_9_N_4_OS_2_)^+^, 214 (C_6_H_5_N_4_OS­_2_)^+^, 195 (C_10_H_13_NOS)^+^, 113 (C_4_H_5_N_2_S)^+^, 106 (C_7_H_8_N)^ +^, 55 (C_3_H_3_O)^+^.

*3-({5-[(2-Amino-1,3-thiazol-4-yl)methyl]-1,3,4-oxadiazol-2-yl}sulfanyl)-N-(3-methylphenyl)propanamide (* ***7c*** *)*

Light brown solid; Yield: 82%; m.p.: 171-172 °C; Mol. Formula: C_16_H_17_N_5_O_2_S_2_; Mol. Mass: 375 gmol^-1^; IR (KBr, *ʋ*/cm^-1^): 3350 (N-H stretching), 3052 (C-H of aromatic ring), 2923 (-CH_2_- stretching), 1670 (C=O stretching), 1641 (C=N stretching), 1576 (C=C stretching of aromatic ring); ^1^H-NMR (DMSO-d_6_, 600 MHz, *δ*/ppm): 9.48 (s, 1H, -NH-CO-1’’), 7.32 (br.d, *J* = 7.5 Hz, 1H, H-6’’’), 7.29 (br.s, 1H, H-2’’’), 7.25-7.23 (m, 2H, H-4’’’, H-5’’’), 6.99 (br.s, 2H, -NH_2_), 6.43 (s, 1H, H-5), 4.06 (br.s, 2H, CH_2_-6), 3.46 (br.t, 2H, *J* = 6.7 Hz, CH_2_-3’’), 2.91 (br.t, 2H, *J* = 6.7 Hz, CH_2_-2’’), 2.25 (br.s, 3H, 3’’’-CH_3_); ^13^C-NMR (DMSO-d_6_, 150 MHz, *δ*/ppm): 168.73 (C-1’’), 168.69 (C-5’), 165.66 (C-2’), 163.41 (C-2), 143.90 (C-4), 138.01 (C-1’’’), 137.62 (C-3’’’), 128.68 (5’’’), 123.94 (C-4’’’), 119.64 (C-6’’’), 116.28 (C-2’’’), 103.17 (C-5), 35.66 (C-6), 27.87 (C-2’’), 27.42 (C-3’’), 21.12 (3’’’-CH_3_); EI-MS: m/z 375 [M]^+^, 241 (C_8_H_9_N_4_OS_2_)^+^, 214 (C_6_H_5_N_4_OS_2_)^+^, 195 (C_10_H_13_NOS)^+^, 113 (C_4_H_5_N_2_S)^+^, 107 (C_7_H_9_N)^+^, 91 (C_7_H_7_)^+^, 55 (C_3_H_3_O)^+^.

*3-({5-[(2-Amino-1,3-thiazol-4-yl)methyl]-1,3,4-oxadiazol-2-yl}sulfanyl)-N-(4-methylphenyl)propanamide (* ***7d*** *)*

Light brown solid; Yield: 80 %; m.p.: 177-178 °C; Mol. Formula: C_16_H_17_N_5_O_2_S_2_; Mol. Mass: 375 gmol^-1^; IR (KBr, *ʋ*/cm^-1^): 3360 (N-H stretching), 3050 (C-H of aromatic ring), 2923 (-CH_2_- stretching), 1666 (C=O stretching), 1643 (C=N stretching), 1580 (C=C stretching of aromatic ring); ^1^H-NMR (DMSO-d_6_, 600 MHz, *δ*/ppm): 9.95 (s, 1H, -NH-CO-1’’), 7.45 (br.d, *J *= 8.4 Hz, 2H, H-2’’’ and H-6’’’), 7.11 (br.d, *J *= 8.3 Hz, 2H, H-3’’’ and H-5’’’), 7.00 (br.s, 2H, -NH_2_), 6.42 (s, 1H, H-5), 4.05 (br.s, 2H, CH_2_-6), 3.46 (br.t, 2H, *J* = 6.7, CH_2_-3’’), 2.86 (br.t, 2H, *J* = 6.7, CH_2_-2’’), 2.25 (s, 3H, 4’’’-CH_3_); ^13^C-NMR (DMSO-d_6_, 150 MHz, *δ*/ppm): 168.71 (C-1’’), 168.40 (C-5’), 165.66 (C-2’), 163.40 (C-2), 143.94 (C-4), 136.39 (C-1’’’), 132.10 (C-4’’’), 129.05 (C-3’’’ and C-5’’’), 119.06 (C-2’’’ and C- 6’’’), 103.15 (C-5), 35.59 (C-6), 27.71 (C-2’’), 27.51 (C-3’’), 20.39 (4’’’-CH_3_); EI-MS: m/z 375 [M]^+^, 269 (C_9_H_9_N_4_O_2_S_2_)^+^, 241 (C_8_H_9_N_4_OS_2_)^+^, 214 (C_6_H_5_N_4_OS­_2_)^+^, 195 (C_10_H_13_NOS)^ +^, 113 (C_4_H_5_N_2_S)^+^, 107 (C_7_H_9_N)^ +^, 55 (C_3_H_3_O)^+^.

*3-({5-[(2-Amino-1,3-thiazol-4-yl)methyl]-1,3,4-oxadiazol-2-yl}sulfanyl)-N-(2,4-dimethylphenyl)propanamide (* ***7e*** *)*

Light brown solid; Yield: 80%; m.p.: 171-172 °C; Mol. Formula: C_17_H_19_N_5_O_2_S_2_; Mol. Mass: 389 gmol^-1^; IR (KBr, *ʋ*/cm^-1^): 3360 (N-H stretching), 3045 (C-H of aromatic ring), 2923 (-CH_2_- stretching), 1668 (C=O stretching), 1647 (C=N stretching), 1546 (C=C stretching of aromatic ring); ^1^H-NMR (DMSO-d_6_, 600 MHz, *δ*/ppm): 9.95 (s, 1H, -NH-CO-1’’), 7.23 (br.d, *J *= 7.5 Hz, 1H, H-6’’’), 7.04 (br.s, 1H H-3’’’), 7.01 (br.s, 2H, -NH_2_), 6.94 (dist.d, *J *= 7.6 Hz, 1H, H-5’’’), 6.42 (s, 1H, H-5), 4.05 (br.s, 2H, CH_2_-6), 3.48 (br.t, 2H, *J* = 6.7 Hz, CH_2_-3’’), 2.89 (br.t, 2H, *J* = 6.7 Hz, CH_2_-2’’), 2.23 (br.s, 3H, 4’’’-CH_3_), 2.12 (br.s, 3H, 2’’’-CH_3_); ^13^C-NMR (DMSO-d_6_, 150 MHz, *δ*/ppm): 168.72 (C-1’’), 168.68 (C-5’), 165.66 (C-2’), 163.40 (C-2), 143.90 (C-4), 134.26 (C-1’’’), 133.47 (C-2’’’), 131.65 (C-4’’’), 130.74 (C-5’’’), 126.35 (C-3’’’), 125.07 (C-6’’’), 103.17 (C-5), 35.66 (C-6), 27.68 (C-2’’), 27.52 (C-3’’), 20.42 (4’’’-CH_3_), 17.76 (2’’’-CH_3_); EI-MS: m/z 375 [M]^+^, 269 (C_9_H_9_N_4_O_2_S_2_)^+^, 241 (C_8_H_9_N_4_OS_2_)^+^, 214 (C_6_H_5_N_4_OS­_2_)^+^, 195 (C_10_H_13_NOS)^+^, 121 (C_8_H_11_N)^+^, 106 (C_8_H_10_), 55 (C_3_H_3_O)^+^.

*3-({5-[(2-Amino-1,3-thiazol-4-yl)methyl]-1,3,4-oxadiazol-2-yl}sulfanyl)-N-(2,5-dimethylphenyl)propanamide (* ***7f*** *)*

Dull white solid; Yield: 89 %; m.p.: 134-135 °C; Mol. Formula: C_17_H_19_N_5_O_2_S_2_; Mol. Mass: 389 gmol^-1^; IR (KBr, *ʋ*/cm^-1^): 3345 (N-H stretching), 3050 (C-H of aromatic ring), 2923 (-CH_2_- stretching), 1666 (C=O stretching), 1641 (C=N stretching), 1546 (C=C stretching of aromatic ring); ^1^H-NMR (DMSO-d_6_, 600 MHz, *δ*/ppm): 9.40 (s, 1H, -NH-CO-1’’), 7.59 (dist.s, 1H, H-6’’’), 7.30 (br.d, *J* = 7.1 Hz, 1H, H-3’’’), 7.06 (br.d, *J* = 7.2 Hz, 1H, H-4’’’), 7.01 (br.s, 2H, -NH_2_), 6.42 (s, 1H, H-5), 4.05 (br.s, 2H, CH_2_-6), 3.48 (br.t, 2H, *J* = 6.7 Hz, CH_2_-3’’), 2.89 (br.t, 2H, *J* = 6.7 Hz, CH_2_-2’’), 2.18 (br.s, 3H, 5’’’-CH_3_), 2.16 (br.s, 3H, 2’’’-CH_3_); ^13^C-NMR (DMSO-d_6_, 150 MHz, *δ*/ppm): 168.73 (C-1’’), 168.68 (C-5’), 165.66 (C-2’), 163.40 (C-2), 143.90 (C-4), 137.99 (C-1’’’), 131.62 (5’’’), 130.61 (C-2’’’), 126.65 (C-3’’’), 123.23 (C-4’’’), 119.05 (C-6’’’), 103.17 (C-5), 35.66 (C-6), 27.68 (C-2’’), 27.52 (C-3’’), 19.60 (5’’’-CH_3_), 18.80 (2’’’-CH_3_); EI-MS: m/z 389 [M]^+^, 269 (C_9_H_9_N_4_O_2_S_2_)^+^, 241 (C_8_H_9_N_4_OS_2_)^+^, 214 (C_6_H_5_N_4_OS­_2_)^+^, 175 (C_11_H_13_NO)^+^, 120 (C_8_H_10_N)^+^, 106 (C_8_H_10_), 55 (C_3_H_3_O)^+^.

*3-({5-[(2-Amino-1,3-thiazol-4-yl)methyl]-1,3,4-oxadiazol-2-yl}sulfanyl)-N-(2,6-dimethylphenyl)propanamide (* ***7g*** *)*

Light brown solid; Yield: 87%; m.p.: 149-150 °C; Mol. Formula: C_17_H_19_N_5_O_2_S_2_; Mol. Mass: 389 gmol^-1^; IR (KBr, *ʋ/*cm^-1^): 3360 (N-H stretching), 3020 (C-H of aromatic ring), 2923 (-CH_2_- stretching), 1667 (C=O stretching), 1651 (C=N stretching), 1580 (C=C stretching of aromatic ring); ^1^H-NMR (DMSO-d_6_, 600 MHz, *δ*/ppm): 9.37 (s, 1H, -NH-CO-1’’), 7.95 (br.s, 2H, -NH_2_), 7.05 (dist.d, *J* = 6.9 Hz, 2H, H-3’’’ and H-5’’’), 6.98 (dist.s, H-4’’’), 6.42 (s, 1H, H-5), 4.05 (br.s, 2H, CH_2_-6), 3.48 (br.t, 2H, *J* = 6.6 Hz, CH_2_-3’’), 2.89 (br.t, 2H, *J* = 6.6 Hz, CH_2_-2’’), 2.12 (br.s, 6H, 2’’’-CH_3_ and 6’’’-CH_3_); ^13^C-NMR (DMSO-d_6_, 150 MHz, *δ*/ppm): 168.72 (C-1’’), 168.29 (C-5’), 165.65 (C-2’), 163.41 (C-2), 143.94 (C-4), 135.06 (C-2’’’ and C-6’’’), 134.91 (C-1’’’), 127.62 (C-3’’’ & C-5’’’), 126.40 C-4’’’), 103.17 (C-5), 34.62 (C-6), 28.11 (C-2’’), 27.52 (C-3’’), 18.09 (2’’’-CH_3_ & 6’’’-CH_3_); EI-MS: m/z 389 [M]^+^, 269 (C_9_H_9_N_4_O_2_S_2_)^+^, 241 (C_8_H_9_N_4_OS_2_)^+^, 214 (C_6_H_5_N_4_OS­_2_)^+^, 175 (C_11_H_13_NO)^+^, 121 (C_8_H_11_N)^+^, 106 (C_8_H_10_), 55 (C_3_H_3_O)^+^.

*3-({5-[(2-Amino-1,3-thiazol-4-yl)methyl]-1,3,4-oxadiazol-2-yl}sulfanyl)-N-(3,4-dimethylphenyl)propanamide (* ***7h*** *)*

Dark brown solid; Yield: 81%; m.p.: 177-178 °C; Mol. Formula: C_17_H_19_N_5_O_2_S_2_; Mol. Mass: 389 gmol^-1^; IR (KBr, *ʋ*/cm^-1^): 3350 (N-H stretching), 3052 (C-H of aromatic ring), 2923 (-CH_2_- stretching), 1669 (C=O stretching), 1648 (C=N stretching), 1570 (C=C stretching of aromatic ring); ^1^H-NMR (DMSO-d_6_, 600 MHz, *δ*/ppm): 9.96 (s, 1H, -NH-CO-1’’), 7.27 (br.d, 1H, *J* = 2.1 Hz, H-2’’’), 7.22 (dd, 1H, *J* = 2.2, 8.1 Hz, H-5’’’), 7.04 (br.d, 1H, *J* = 8.1 Hz, H-6’’’), 7.00 (br.s, 2H, -NH_2_), 6.41 (s, 1H, H-5), 4.05 (br.s, 2H, CH_2_-6), 3.46 (br.t, 2H, *J* = 6.7 Hz, CH_2_-3’’), 2.85 (br.t, 2H, *J* = 6.7 Hz, CH_2_-2’’), 2.20 (br.s, 3H, 4’’’-CH_3_), 2.16 (br.s, 3H, 3’’’-CH_3_); ^13^C-NMR (DMSO-d_6_, 150 MHz, *δ*/ppm): 168.71 (C-1’’), 168.63 (C-5’), 165.65 (C-2’), 163.39 (C-2), 143.94 (C-4), 136.70 (C-1’’’), 136.62 (C-3’’’), 131.98 (C-4’’’), 126.35 (C-5’’’), 120.49 (C-2’’’), 120.31 (C- 6’’’), 103.14 (C-5), 35.57 (C-6), 27.73 (C-2’’), 27.51 (C-3’’), 19.59 (3’’’-CH_3_), 18.75 (4’’’-CH_3_); EI-MS: m/z 389 [M]^+^, 269 (C_9_H_9_N_4_O_2_S_2_)^+^, 241 (C_8_H_9_N_4_OS_2_)^+^, 214 (C_6_H_5_N_4_OS­_2_)^+^, 175 (C_11_H_13_NO)^+^, 120 (C_8_H_10_N)^+^, 106 (C_8_H_10_), 55 (C_3_H_3_O)^+^.

*3-({5-[(2-Amino-1,3-thiazol-4-yl)methyl]-1,3,4-oxadiazol-2-yl}sulfanyl)-N-(3,5-dimethylphenyl)propanamide (* ***7i*** *)*

Light brown solid; Yield: 82%; m.p.: 180-181 °C; Mol. Formula: C_17_H_19_N_5_O_2_S_2_; Mol. Mass: 389 gmol^-1^; IR (KBr, *ʋ*/cm^-1^): 3350 (N-H stretching), 3050 (C-H of aromatic ring), 2920 (-CH_2_- stretching), 1667 (C=O stretching), 1651 (C=N stretching), 1580 (C=C stretching of aromatic ring); ^1^H-NMR (DMSO-d_6_, 600 MHz, *δ*/ppm): 9.89 (s, 1H, -NH-CO-1’’), 7.21 (dist.s, 2H, H-2’’’ and H-6’’’), 7.19 (br.s, 2H, -NH_2_), 6.98 (br.s, H-4’’’), 6.42 (s, 1H, H-5), 4.05 (br.s, 2H, CH_2_-6), 3.48 (br.t, 2H, *J* = 6.6 Hz, CH_2_-3’’), 2.88 (br.t, *J* = 6.6 Hz, CH_2_-2’’), 2.12 (br.s, 6H, 3’’’-CH_3_ and 5’’’-CH_3_); ^13^C-NMR (DMSO-d_6_, 150 MHz, *δ*/ppm): 168.72 (C-1’’), 168.29 (C-5’), 165.65 (C-2’), 163.41 (C-2), 143.94 (C-4), 137.65 (C-1’’’), 135.06 (C-3’’’ and C-5’’’), 127.62 (C-4’’’), 124.34 (C-2’’’ and C-6’’’), 103.17 (C-5), 35.62 (C-6), 28.11 (C-2’’), 27.52 (C-3’’), 21.06 (3’’’-CH_3_ and 5’’’-CH_3_); EI-MS: m/z 389 [M]^+^, 269 (C_9_H_9_N_4_O_2_S_2_)^+^, 241 (C_8_H_9_N_4_OS_2_)^+^, 214 (C_6_H_5_N_4_OS­_2_)^+^, 175 (C_11_H_13_NO)^+^, 121 (C_8_H_11_N)^+^, 106 (C_8_H_10_), 55 (C_3_H_3_O)^+^.

*3-({5-[(2-Amino-1,3-thiazol-4-yl)methyl]-1,3,4-oxadiazol-2-yl}sulfanyl)-N-(2-ethylphenyl)propanamide (* ***7j*** *)*

Light brown solid; Yield: 91%; m.p.: 198-199 °C; Mol. Formula: C_17_H_19_N_5_O_2_S_2_; Mol. Mass: 389 gmol^-1^; IR (KBr, *ʋ*/cm^-1^): 3350 (N-H stretching), 3075 (C-H of aromatic ring), 2930 (-CH_2_- stretching), 1667 (C=O stretching), 1650 (C=N stretching), 1576 (C=C stretching of aromatic ring); ^1^H-NMR (DMSO-d_6_, 600 MHz, *δ*/ppm): *δ* 9.42 (s, 1H, -NH-CO-1’’), 7.32 (dist.dd, 1H,* J* = 1.3, 7.2 Hz, H-6’’’), 7.22 (dd, *J* = 1.5, 7.1 Hz, 1H, H-3’’’), 7.17-7.12 (m, 2H, H-4’’’ and H-5’’’), 6.99 (br.s, 2H, -NH_2_), 6.41 (s, 1H, H-5), 4.05 (br.s, 2H, CH_2_-6), 3.47 (br.t,* J* = 6.7 Hz, 2H, CH_2_-3’’), 2.90 (br.t,* J* = 6.7 Hz, CH_2_-2’’), 2.56 (q, *J* = 7.6 Hz, 2H, 2’’’-CH_2_-CH_3_), 1.09 (t, 3H, *J* = 7.6 Hz, 2’’’-CH_2_-CH_3_); ^13^C-NMR (DMSO-d_6_, 150 MHz, *δ*/ppm): 168.99 (C-5’), 168.71 (C-1’’), 165.65 (C-2’), 163.42 (C-2), 143.94 (C-4), 137.91 (C-1’’’), 135.30 (C-2’’’), 128.48 (C-3’’’), 126.03 (C-5’’’), 125.84 (C-4’’’), 125.71 (C-6’’’), 103.15 (C-5), 35.01 (C-6), 27.97 (C-2’’), 27.52 (C-3’’), 23.72 (2’’’-CH_2_-CH_3_), 14.21 (2’’’-CH_2_-CH_3_); EI-MS: m/z 389 [M]^+^, 269 (C_9_H_9_N_4_O_2_S_2_)^+^, 241 (C_8_H_9_N_4_OS_2_)^+^, 214 (C_6_H_5_N_4_OS­_2_)^+^, 175 (C_11_H_13_NO)^+^, 121 (C_8_H_11_N)^+^, 106 (C_8_H_10_), 55 (C_3_H_3_O)^+^.

*3-({5-[(2-amino-1,3-thiazol-4-yl)methyl]-1,3,4-oxadiazol-2-yl}sulfanyl)-N-(4-ethylphenyl)propanamide (* ***7k*** *)*

Light brown solid; Yield: 84 %; m.p.: 193-194 °C; Mol. Formula: C_17_H_19_N_5_O_2_S_2_; Mol. Mass: 389 gmol^-1^; IR (KBr, *ʋ*/cm^-1^): 3345 (N-H stretching), 3062 (C-H of aromatic ring), 2923 (-CH_2_- stretching), 1663 (C=O stretching), 1649 (C=N stretching), 1576 (C=C stretching of aromatic ring); ^1^H-NMR (DMSO-d_6_, 600 MHz, *δ*/ppm): 9.96 (s, 1H, -NH-CO-1’’), 7.47 (br.d, *J *= 8.5 Hz, 2H, H-2’’’ and H-6’’’), 7.13 (br.d, *J *= 8.5 Hz, 2H, H-3’’’ and H-5’’’), 6.99 (br.s, 2H, -NH_2_), 6.41 (s, 1H, H-5), 4.04 (br.s, 2H, CH_2_-6), 3.45 (br.t, 2H, *J* = 6.7, CH_2_-3’’), 2.86 (br.t, 2H, *J* = 6.7, CH_2_-2’’), 2.54 (q, 2H, *J* = 7.5 Hz, 4’’’-CH_2_-CH_3_), 1.14 (t, 3H, *J* = 7.5 Hz, 4’’’-CH_2_-CH_3_); ^13^C-NMR (DMSO-d_6_, 150 MHz, *δ*/ppm): 168.71 (C-1’’), 168.41 (C-5’), 165.66 (C-2’), 163.40 (C-2), 143.94 (C-4), 138.59 (C-1’’’), 136.58 (C-4’’’), 127.86 (C-3’’’ and C-5’’’), 119.14 (C-2’’’ and C-6’’’), 103.15 (C-5), 35.59 (C-6), 27.72 (C-2’’), 27.54 (4’’’-CH_2_-CH_3_), 27.51 (C-3’’), 15.64 (4’’’-CH_2_-CH_3_); EI-MS: m/z 389 [M]^+^, 269 (C_9_H_9_N_4_O_2_S_2_)^+^, 241 (C_8_H_9_N_4_OS_2_)^+^, 214 (C_6_H_5_N_4_OS­_2_)^+^, 175 (C_11_H_13_NO)^+^, 121 (C_8_H_11_N)^+^, 106 (C_8_H_10_), 55 (C_3_H_3_O)^+^.

*3-({5-[(2-Amino-1,3-thiazol-4-yl)methyl]-1,3,4-oxadiazol-2-yl}sulfanyl)-N-(4-ethoxyphenyl)propanamide (* ***7l*** *)*

Light brown solid; Yield: 80%; m.p.: 177-178 °C; Mol. Formula: C_16_H_17_N_5_O_2_S_2_; Mol. Mass: 375 gmol^-1^; IR (KBr, *ʋ*/cm^-1^): 3360 (N-H stretching), 3050 (C-H of aromatic ring), 2923 (-CH_2_- stretching), 1663 (C=O stretching), 1651 (C=N stretching), 1580 (C=C stretching of aromatic ring); ^1^H-NMR (DMSO-d_6_, 600 MHz, *δ*/ppm): 9.88 (s, 1H, -NH-CO-1’’), 7.46 (br.d, *J *= 9.0 Hz, 2H, H-2’’’ and H-6’’’), 6.99 (br.s, 2H, -NH_2_), 6.85 (br.d, *J *= 9.0 Hz, 2H, H-3’’’ and H-5’’’), 6.41 (s, 1H, H-5), 4.04 (br.s, 2H, CH_2_-6), 3.97 (q, *J* = 6.9 Hz, 2H, 4’’’-OCH_2_-CH_3_), 3.45 (br.t, 2H, *J* = 6.7 Hz, CH_2_-3’’), 2.84 (br.t, 2H, *J* = 6.7 Hz, CH_2_-2’’), 1.29 (t, *J* = 6.9 Hz, 3H, 4’’’-OCH_2_-CH_3_); ^13^C-NMR (DMSO-d_6_, 150 MHz, *δ*/ppm): 168.64 (C-1’’), 168.00 (C-5’), 165.58 (C-2’), 163.33 (C-2), 154.34 (C-4’’’), 143.87 (C-4), 131.89 (C-1’’’), 120.50 (C-2’’’ and C-6’’’), 114.27 (C-3’’’ and C-5’’’), 103.07 (C-5), 62.96 (4’’’-OCH_2_-CH_3_) 35.42 (C-6), 27.71 (C-2’’), 27.44 (C-3’’), 14.57 (4’’’-OCH_2_-CH_3_); EI-MS: m/z 375 [M]^+^, 269 (C_9_H_9_N_4_O_2_S_2_)^+^, 241 (C_8_H_9_N_4_OS_2_)^+^, 214 (C_6_H_5_N_4_OS­_2_)^+^, 136 (C_8_H_10_NO)^+^, 121 (C_8_H_9_O), 55 (C_3_H_3_O)^+^.

*Urease inhibition assay*

This enzyme assay is the customized form of the commonly known Berthelot assay (23, 24). The assay mixture of 85 µL is prepared containing 10 µL of phosphate buffer of pH 7.0 (in each well in the 96-well plate), 10 µL of sample solution and 25 µL of enzyme solution (0.135 units). The contents were pre-incubated at 37 ºC for 5 min. 40 µL of urea stock solution (20 mM) was added to each well with incubation for 10 min at 37 ºC. It is followed by the addition of 115 µL phenol hypochlorite reagents (freshly prepared by mixing 45 µL phenol with 70 µL of alkali) per well. For color development, incubation was carried out for further 10 min at 37 ºC. The absorbance was measured at 625 nm. The percentage enzyme inhibition and IC_50_ values were calculated using the following formula:

$\mathrm{inhibition}\left(\%\right)=\frac{\mathrm{Control}-\mathrm{test}}{\mathrm{Control}}\times 100$

Where, Control is the total enzyme activity without inhibitor and Test is the activity in the presence of test compound. IC_50_ values were calculated using the EZ–Fit Enzyme kinetics software (Perrella Scientific Inc. Amherst, US).

*Statistical analysis*

Statistical analysis was performed by Microsoft Excel 2010 for all the thrice measured values and the results are presented as mean ± SEM.

*Hemolytic activity*

Bovine blood samples was collected in EDTA, that was diluted with saline (0.9% NaCl), and centrifuge at 1000 ×g for 10 min. The erythrocytes separated diluted in phosphate buffer saline of pH 7.4 and a suspension was made. Add 20 µL of synthetic compounds solution (10 mg/mL) in 180 µL of RBCs suspension and incubate for 30 min at room temperature. PBS was used as negative control and Triton 100-X was taken as positive control (25, 26). The percentage of hemolysis was taken as by using Equation:

$\mathrm{Hemolysis}\left(\%\right)=\frac{\mathrm{Absorbance of sample}-\mathrm{Absorbance of negative control}}{\mathrm{Absorbance of positive control}}\times 100$

*Molecular docking*

Initially, the synthesized chemical ligands (**7a-l**) were drawn in ACD/ChemSketch tool and retrieved in mol format. Furthermore, UCSF Chimera 1.10.1 tool was employed for energy minimization having default parameters such as steepest descent steps 100 with step size 0.02 (Å), conjugate gradient steps 100 with step size 0.02 (Å) and update interval was fixed at 10. Finally, Gasteiger charges were assigned in ligands using Dock Prep to obtain the good structure conformation. Molecular docking experiment was employed on **7a-l**, against Jack bean urease by using virtual screening tool PyRx with VINA Wizard approach (27). The grid box parameters values in VINA search space (X = 11.06, Y = -54.61 and Z = -27.12) were adjusted with default exhaustiveness value = 8 to maximize the binding conformational analysis. We have adjusted sufficient grid box size on biding pocket residues to allow the ligand to move freely in the search space. The generated docked complexes were evaluated on the basis of lowest binding energy (kcal/mol) values and binding interaction pattern between ligands and receptor. The graphical depictions of all the docked complexes were accomplished by UCSF Chimera 1.10.1 (28) and Discovery Studio (2.1.0), respectively.

## Results and Discussion

*Chemistry*

**Scheme 1:**
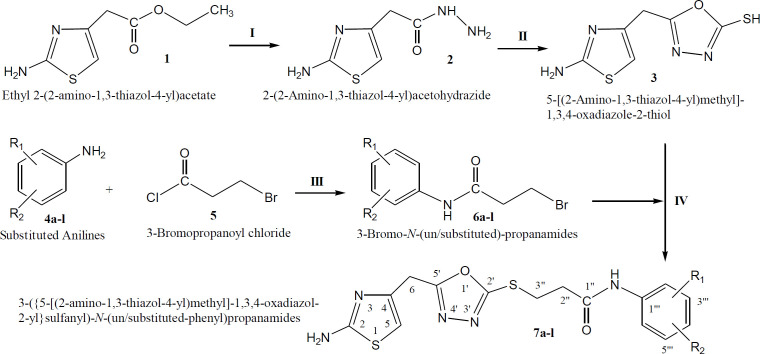
Outline for the synthesis of 3-({5-[(2-amino-1,3-thiazol-4-yl)methyl]-1,3,4-oxadiazol-2-yl}sulfanyl)-*N*-(un/substituted-phenyl)propanamides. Reagents and Conditions: (I) MeOH/N_2_H_4_**·**H_2_O/refluxing for 2 h. (II) EtOH/CS_2_/KOH/refluxing for 5 h. (III) Aq. 10% Na_2_CO_3_ soln./vigorous manual shaking and stirring at RT for 2-3 h. (IV) DMF/LiH/stirring for 3-5

The bi-heterocyclic propanamides were synthesized through a protocol depicted in Scheme 1 and different varying groups are listed in Table 1. The molecules were screened against urease to ascertain their enzyme inhibitory potential. The percent inhibition and IC_50_ values are given in Table 2. The cytotoxicity of these compounds was also evaluated through hemolytic activity and the results of percentage hemolysis are also tabulated in Table 2.

The synthesis of these targeted molecules was accomplished in several steps. Ethyl 2-(2-amino-1,3-thiazol-4-yl)acetate (**1**) was refluxed with hydrazine hydrate in methanol to acquire corresponding hydrazide, **2**. This nucleophillic substitution reaction was completed in two hours. The hydrazide, **2**, was made to react with carbon disulfide in the presence of KOH, an activator for cyclization, to yield a thiol containing nucleophile, 5-[(2-amino-1,3-thiazol-4-yl)methyl]-1,3,4-oxadiazol-2-thiol (**3**). Different electrophiles, **6a-l**, were synthesized by stirring un/substituted anilines (**4a-l)** with 3-bromopropanoyl chloride (**5**) in a weak basic aqueous medium. Finally, the designed bi-heterocyclic propanamides (**7a-l**), were acquired by stirring **3 **with newly synthesized different electrophiles, **6a-l**, in DMF using LiH as a base and an activator. The molecular structures of these derivatives were corroborated by IR, EI-MS, ^1^H-NMR and ^13^C-NMR spectral data. 

**Figure 1:**
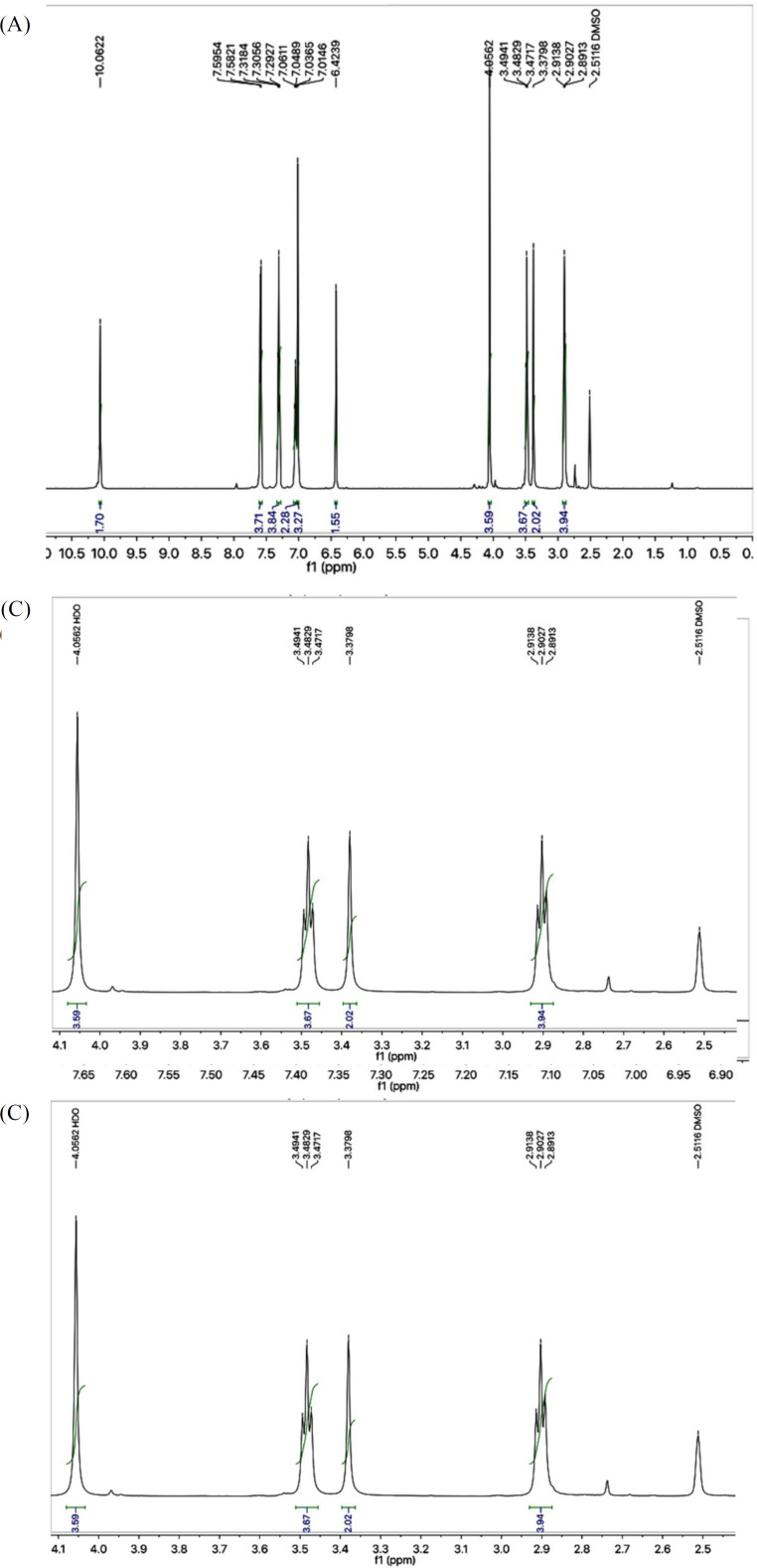
(A) ^1^H-NMR spectrum of **7a**. (B) Expanded aromatic region of ^1^H-NMR spectrum of **7a**. (C) Expanded aliphatic region of ^1^H-NMR spectrum of **7a**

The structural characterization of one the compounds is discussed hereby in detail for the benefit of the reader. Compound **7a **was obtained as a light brown solid with 89% yield. Its molecular formula, C_15_H_15_N_5_O_2_S_2_, was recognized through molecular ion peak at m/z 361 in its EI-MS spectrum. Counting the number of protons in its ^1^H-NMR spectrum (Figure 1A) and number of carbon resonances in its ^13^C-spectrum was also supportive in assigning its molecular formula. Various functionalities in the molecule were identified through its IR spectrum and prominent absorption bands appeared at *ʋ* 3355 (N-H stretching), 3050 (C-H of aromatic ring), 2923 (-CH_2_- stretching), 1665 (C=O stretching), 1645 (C=N stretching), and 1577 (C=C stretching of aromatic ring) cm^-1^. The phenyl group attached with nitrogen of amide functionality was clearly depicted by three signals in aromatic region at *δ* 7.59 (br.d, 2H, *J* = 7.9 Hz, H-2’’’ and H-6’’’), 7.30 (br.t, *J* = 7.7 Hz, 2H, H-3’’’ and H-5’’’), and 7.04 (br.t, 1H, *J* = 7.4, H-4’’’) (Figure 1B). 2-Amino-1,3-thiazol-4-yl heterocyclic moiety was rationalized by two singlets at *δ* 7.01 (s, 2H, -NH_2_), and 6.42 (s, 1H, H-5) while the propanamide unit in the molecule was specified by a downfield amidic singlet at *δ* 10.06 (s, 1H, -NH-CO-1’’) along with two broad-triplets in aliphatic region at *δ* 3.48 (br.t, 2H, *J* = 6.7, CH_2_-3’’), and 2.89 (br.t, 2H, *J* = 6.7 Hz, CH_2_-2’’), symbolic for two interconnected methylenes (Figure 1C). The signal at *δ* 4.05 (br.s, 2H, CH_2_-6), was assignable to a methylene uniting the two heterocycles (2-amino-1,3-thiazol-4-yl and 1,3,4-oxadiazole) in the molecule. 

**Figure 2:**
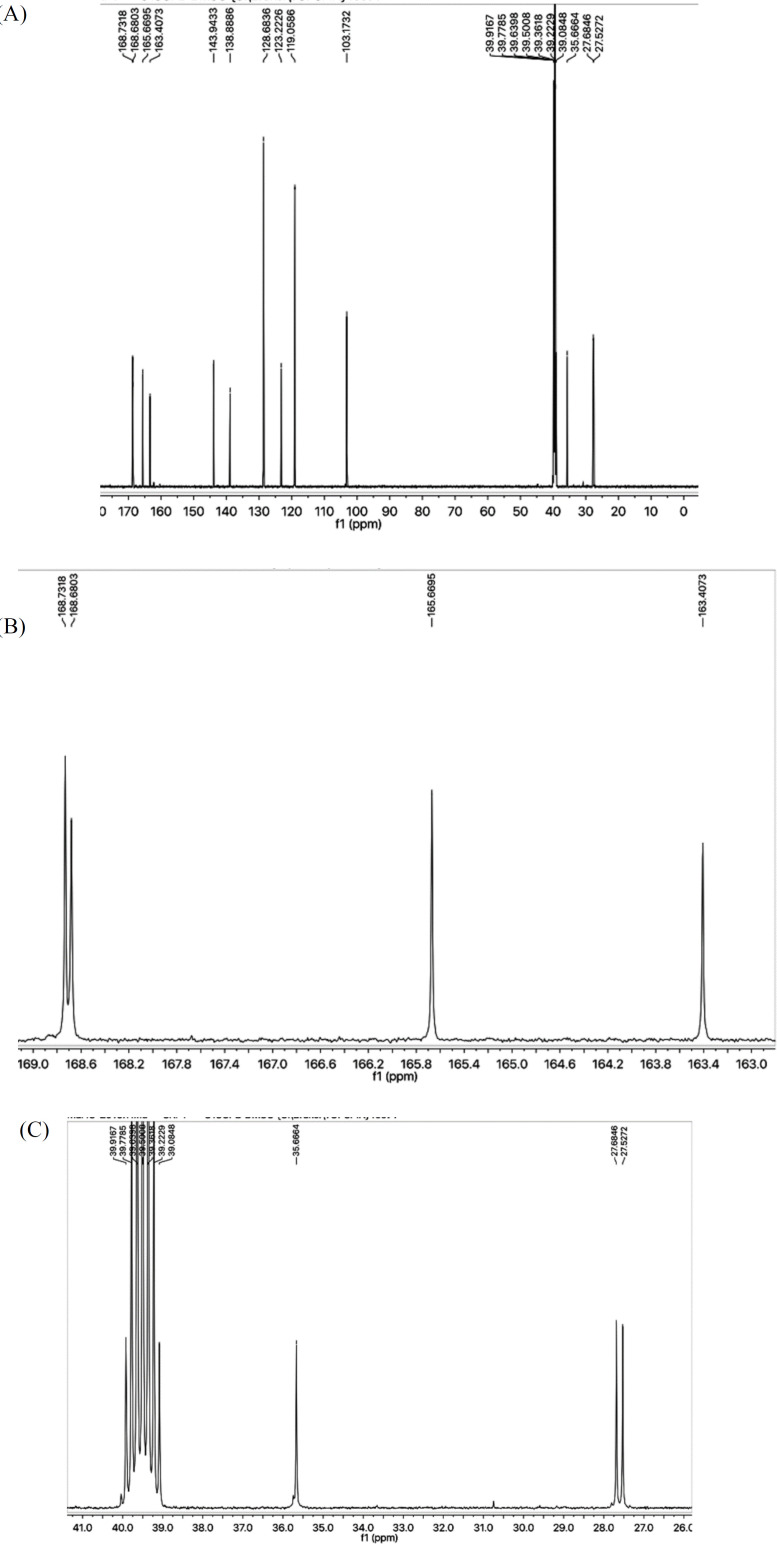
(A) ^13^C-NMR spectrum of **7a**. (B) Expanded downfield region of ^13^C-NMR spectrum of **7a**. (C) Expanded upfield region of ^13^C-NMR spectrum of **7a**

All these assignments were also fully corroborated by its ^13^-C-NMR spectrum (Figure 2A). The *N*-phenyl group was clearly represented by a quaternary signal at *δ* 138.38 (C-1’’’) and three methine signals are *δ* 128.68 (C-3’’’ and 5’’’), 123.22 (C-4’’’), and 119.05 (C-2’’’ and C-6’’’). 2-Amino-1,3-thiazol-4-yl heterocycle was recognized by two quaternary signals at *δ* 163.40 (C-2), and 143.94 (C-4) along with a methine signal at *δ* 103.17 (C-5) while the other heterocycle i.e. 1,3,4-oxadiazole was endorsed with two quaternary resonances at *δ* 168.68 (C-5’), and 165.66 (C-2’) (Figure 2B). The propanamide part in the molecule was clearly indicated by a most downfield quaternary signal for a carbonyl group at *δ* 168.73 (C-1’’) along with two upfield methylene signals at *δ* 27.68 (C-2’’), and 27.52 (C-3’’). Another signal at *δ* 35.66 (C-6) was assignable to a methylene connecting the 2-amino-1,3-thiazol-4-yl heterocycle with 1,3,4-oxadiazole heterocyle via the forth position of former to the fifth position of latter (Figure 2C). 

**Figure 3:**
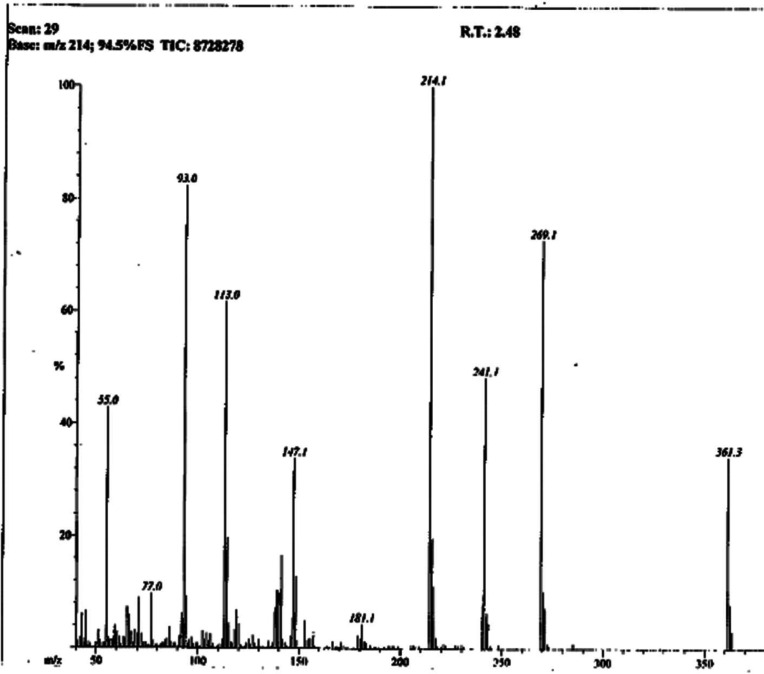
EI-MS spectrum of **7a**

The presence of these moieties was also apparent from various fragment ion peaks in its EI-MS spectrum (Figure 3), as given in the experimental section. 

Based upon these collective evidences, the structure of **7a** was confirmed and it was named as 3-({5-[(2-amino-1,3-thiazol-4-yl)methyl]-1,3,4-oxadiazol-2-yl}sulfanyl)-*N*-phenylpropanamide. A similar approach was implemented for the structural confirmation of other derivatives.

*Urease inhibition and structure-activity relationship*

The synthesized bi-heterocyclic propan-amides were screened against urease and found to have potent inhibitory potential against this enzyme, evident from their much lower IC_50 _(mM) values as compared to the standard thiourea, as tabulated in Table 2. These derivatives demonstrated inhibition in the range of 5.18 ± 0.06 to 1.24 ± 0.01 µM, relative to thiourea having IC_50 _value of 21.11 ± 0.12 µM. So, it was pertinent to say that these molecules possessed many-folds better inhibitory potentials as compared to the standard.

**Figure 4:**
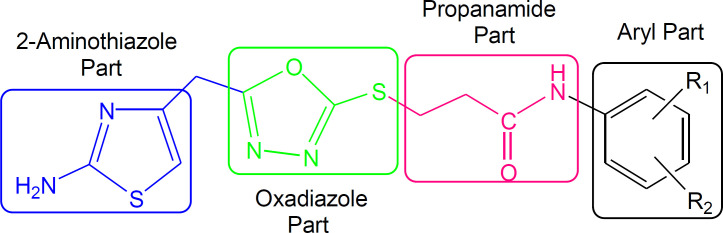
General structural features of compounds **7a-l**

Although the displayed activity is an attribute of a whole molecule, but a limited structure-activity relationship (SAR) was recognized by examining the effect of different groups (-R_1 _& -R_2_) attached to phenyl ring (aryl part) on the inhibitory potential. Figure 4 exposed the general structural features of the studied multifunctional compounds.

**Figure 5:**
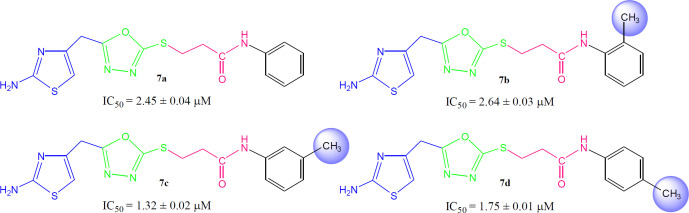
Structure-activity relationship of compounds **7a**, **7b**, **7c**, and **7d**

Compound **7a** (IC_50_ = 2.45 ± 0.04 µM) with unsubstituted phenyl ring (aryl part) exhibited very resembling inhibitory potential with mono-substituted molecule **7b **(IC_50_ = 2.64 ± 0.03 µM) in which a small sized methyl group was present at 2-position. Compound **7c** (IC_50_ = 1.32 ± 0.02 µM) with methyl group at 3-position, and **7d **(IC_50_ = 1.75 ± 0.01 µM) with a methyl group at 4-position; however exhibited somewhat greater inhibitory potential relative to **7a**. Indeed, the **7c** was identified as a second most active compound in the synthetic series. So it was cogent from the present observations that when a small sized group was present at *meta* or *para*-position, the molecule was attributed with superb inhibitory potential (Figure 5).

**Figure 6:**
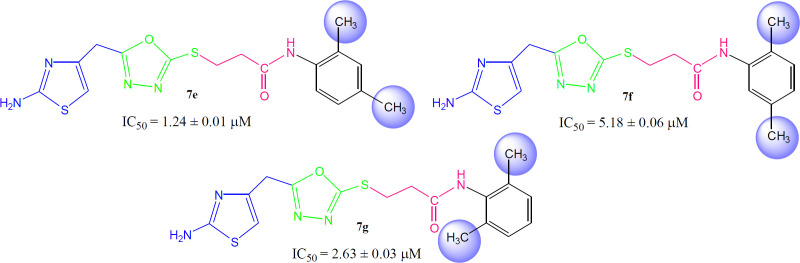
Structure-activity relationship of **7e**, **7f**, and **7g**

However, among the di-methylated regio-isomers, compound **7e** in which two small sized groups were present at 2- and 4-position, possessed a remarkable inhibitory potential (IC_50_ = 1.24 ± 0.01 µM) and was the most active in synthesized series. A decrease in inhibitory potential (IC_50_ = 5.18 ± 0.06 µM) was observed in **7f** in which the methyl groups were present at 2- and 5-position as compared to the isomer **7g** (IC_50_ = 2.63 ± 0.03 µM) in which these groups were present at 2- and 6-position (Figure 6). It means the presence of one of the methyl group on *para*-position in aryl part of such regio-isomers, probably resulted in the superb interaction with the active site of the enzyme. 

**Figure 7:**
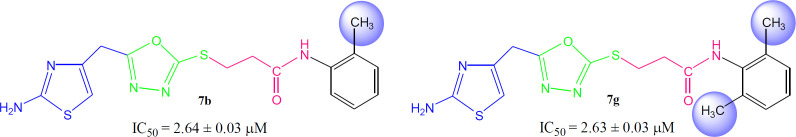
Structure-activity relationship of **7b**, and **7g**

When the inhibitory potential of mono-*ortho* substituted molecule **7b **(IC_50_ = 2.64 ± 0.03 µM) was compared with di-*ortho* substituted molecule **7g **(IC_50_ = 2.63 ± 0.03 µM), it was surprising to know that the additional small sized *ortho*-substituent in latter molecule was not contributing to vary its inhibitory potential as compared to former molecule in a considerable manner (Figure 7). 

**Figure 8:**
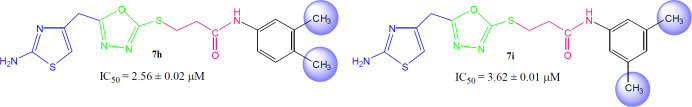
Structure-activity relationship of **7h**, and **7i**

Among the following two analogues, **7h** bearing 3,4-dimethyl residues in aryl part displayed slightly better inhibitory potential (IC_50_ = 2.56 ± 0.02 µM) as compared to that of **7i** (IC_50_ = 3.62 ± 0.01 µM), in which these methyl groups were present at 3- and 5-position (Figure 8). So it was guessed that the presence of one of the substituent again at *para*-position attributed the molecule to have suitable interactions with the enzyme. 

**Figure 9:**
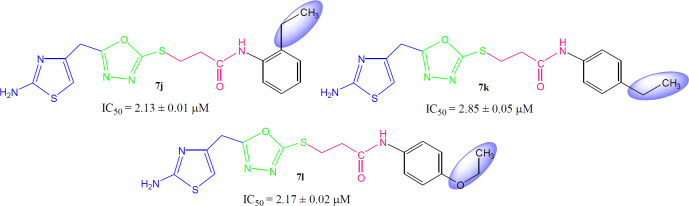
Structure-activity relationship of **7j, 7k**, and **7l**

Instead of small sized groups, when medium sized groups were present in the following three compounds (Figure 9), although their inhibitory potentials were very close, yet a reverse trend was observed on a closer look. Hereby, **7j **with *ortho*-ethyl group behaved as slightly better inhibitor (IC_50_ = 2.13 ± 0.01 µM) as compared to both *para*-substituted molecules, **7k** (IC_50_ = 2.85 ± 0.05 µM) and **7l **(IC_50_ = 2.17 ± 0.02 µM). 

 So, it was postulated, from the structure-activity relationship among these bi-heterocyclic propanamides, that the compound bearing a small sized group at *meta-*position or the di-substituted molecules, bearing at least one small sized group at *para*-position, were generally potent inhibitors of the urease enzyme. However, when a medium sized group was present on aryl part of these molecules, no significant variation in their inhibitory potential was observed. 

Synthesized compounds can be arranged in the following row according to their inhibitory activity: **7d **>** 7c **>** 7d **>** 7j **>** 7l **>** 7a **>** 7h **>** 7g **>** 7b **>** 7k **> **7i** > **7f **(see Table 2 for IC_50_ values).

*Hemolytic activity*

The cytotoxicity of the synthesized compounds was evaluated through hemolytic assay. Results of percentage hemolysis are shown in Table 2 which indicated that all these compounds were approximately nontoxic for membrane of red blood cells and their hemolysis values ranged from 5.16% to 11.47%, which were much lower than the Triton-X (positive control) having percentage hemolysis of 89%.

*Molecular docking analysis*

*Docking energy evaluation of ligands*

To predict the best conformational position within the active region of urease the generated docked complexes were examined on the basis of minimum energy values (kcal/mol) and bonding interaction pattern such as hydrogen and hydrophobic, respectively. Docking results justified that all compounds, **7a-l**, depict good energy values (kcal/mol) (Table 3). The standard error for Autodock is reported as 2.5 kcal/mol (http://autodock.scripps.edu/). The basic nucleus of all the synthesized compounds was unique, therefore most of ligands exhibited good energy values and no higher fluctuated energy score values were observed among all docking complexes. The docking energy calculation is done by Equation 1.

∆Gbinding = ∆Ggauss + ∆Grepulsion + ∆Ghbond + ∆Ghydrophobic + ∆Gtors (Equation 1)

∆G gauss Attractive term for dispersion, two gaussian functions, ∆Grepulsion Square of the distance if closer than a threshold value, ∆G hbond Ramp function - also used for interactions with metal ions, ∆G hydrophobic Ramp function, ∆G tors Proportional to the number of rotatable bonds

*Binding pocket evaluation*

**Figure 10:**
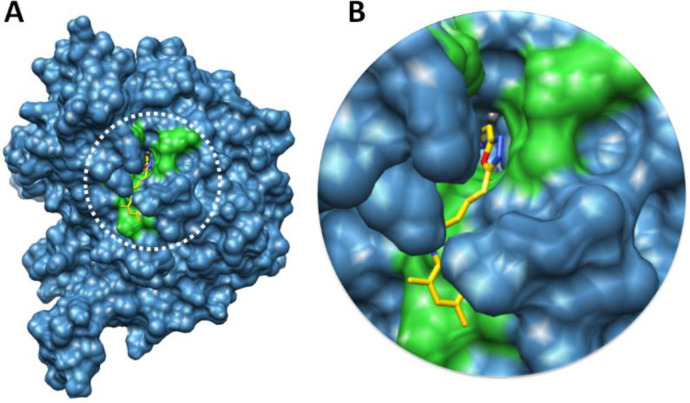
Docking complex **7e** against urease. (A) The urease structure is highlighted in surface format having blue color while the binding pocket is justified in green color in surface format. (B) The closer view of binding pocket which shows the ligand (yellow color) structure and its conformation inside the binding pocket

The binding pocket is present in domain 4 where nickel metals are present (29). Based on *in-vitro* analysis **7e** showed good enzyme inhibition potential therefore, ligand **7e** was selected to check the binding interaction pattern. The **7e**-docking complex showed that ligand structure showed its penetration inside the binding pocket. The 2-aminothiazole part of **7e** showed its infiltration toward nickel metal and adjusted within binding pocket of urease. However, the substituted aryl moiety showed good conformation position in the opening part of binding pocket of target protein (Figure 10).

*Hydrogen bonds analysis between* ***7e*** *and urease*

**Figure 11:**
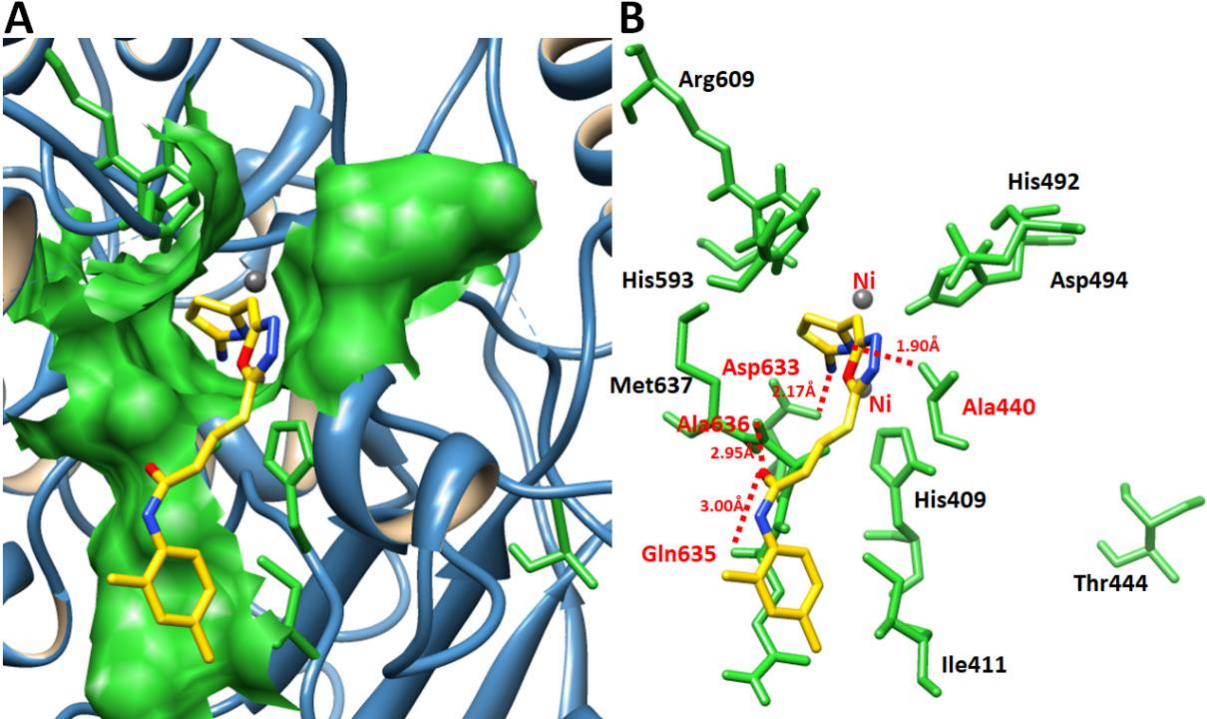
Docking complex of **7e**. (A) The general overview of docking depiction. The protein structure is represented in blue color in ribbon format while ligand is highlighted yellow color. (B) The closer view of binding pocket interaction with best conformation position of ligand **7e** against urease. The ligand molecule is depicted in yellow color while their functional groups such as amino, sulfur, oxygen, and nitrogen are shown in yellow, red, and blue colors, respectively. The binding pocket residues are highlighted in green color. The binding interaction shows in red dotted lines with distances mentioned in angstrom (Å). Two nickel atoms are represented in yellow circles

Figure 11 showed the binding interaction pattern of **7e** against urease. In detail binding analysis it was observed that **7e** forms four hydrogen bonds at different residues of target protein. The free amino group of thiazole ring and nitrogen within the cyclic ring were both prone to make hydrogen bonds. Our results showed that 2-aminothiazole and cyclic nitrogen of **7e** forms two hydrogen bonds against Ala440 and Asp633 having 2.17 and 1.90 Å, respectively. The oxygen of amide group also forms couple of hydrogen bonds with Gln635 and Ala636 with bonds length 2.95 and 3.00 Å, respectively. The docking complex binding pocket residues also showed good correlation with published data which strengthened our docking results (30, 31).

**Table 1:** Different groups (-R_1 _and -R_2_) in Scheme 1.

**Compd.**	**-R**_1_	**-R**_2_
**4a, 6a, 7a**	-H	-H
**4b, 6b, 7b**	2-CH_3_	-H
**4c, 6c, 7c**	3-CH_3_	-H
**4d, 6d, 7d**	-H	4-CH_3_
**4e, 6e, 7e**	2-CH_3_	4-CH_3_
**4f, 6f, 7f**	2-CH_3_	5-CH_3_
**4g, 6g, 7g**	2-CH_3_	6-CH_3_
**4h, 6h, 7h**	3-CH_3_	4-CH_3_
**4i, 6i, 7i**	3-CH_3_	5-CH_3_
**4j, 6j, 7j**	2-CH_2_-CH_3_	-H
**4k, 6k, 7k**	-H	4-CH_2_-CH_3_
**4l, 6l, 7l**	-H	4-O-CH_2_-CH_3_

**Table 2:** Percentage inhibition at 0.5 mM, IC_50_ values for urease and percentage hemolysis

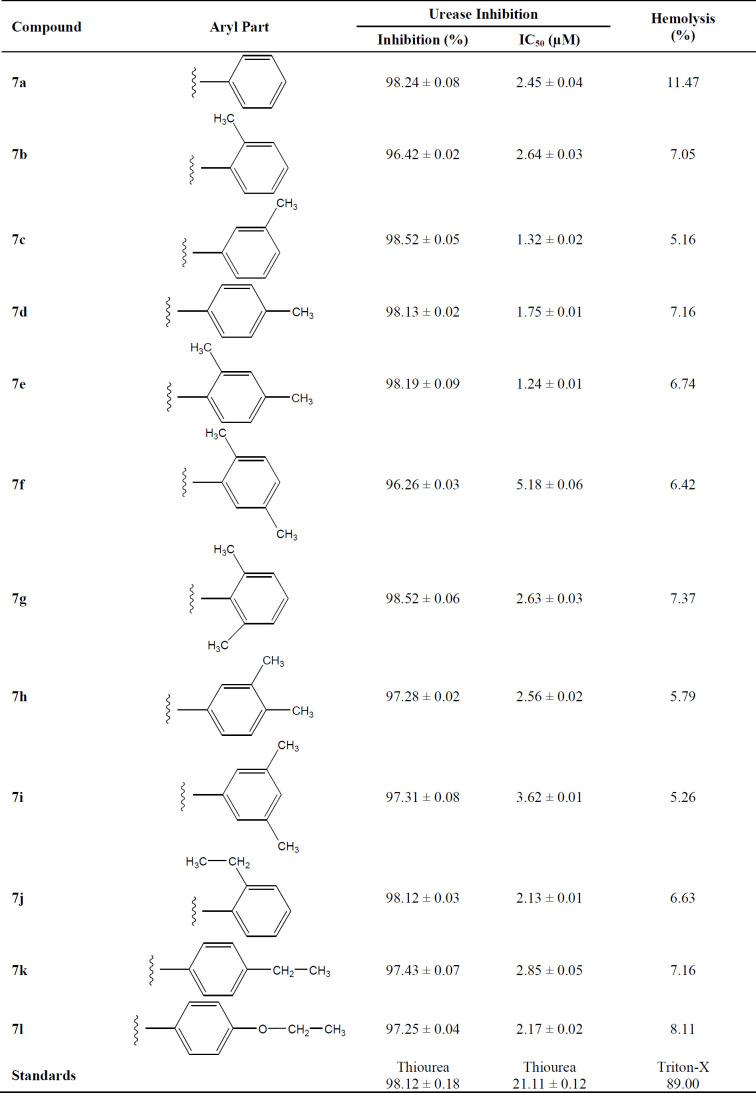

IC_50_ values (concentration at which there is 50% enzyme inhibition) of the compounds were calculated from the inhibition data obtained after doing assays at high dilutions of the compounds as given in assay method and data was computed using EZ–Fit Enzyme kinetics software (Perrella Scientific Inc. Amherst, USA). Data is mean of three values (mean ± SEM, n = 3). PBS Hemolysis = 2.93%.

**Table 3:** The docking energy values ligands, **7a-l**.

**Ligand Complexes**	**Binding Affinity (kcal/mol)**
Recp urease_**7a**	-7.8
Recp urease_**7b**	-7.8
Recp urease_**7c**	-8.8
Recp urease_**7d**	-8.7
Recp urease_**7e**	-9
Recp urease_**7f**	-9.2
Recp urease_**7g**	-8.5
Recp urease_**7h**	-8
Recp urease_**7i**	-8.7
Recp urease_**7j**	-8.7
Recp urease_**7k**	-8.1
Recp urease_**7l**	-7.9

## Conclusion

A series of bi-heterocyclic propanamides was synthesized in appreciable yields. All these molecules demonstrated an excellent inhibitory potential against urease and their *in-vitro* inhibitory results were also coherent with *in-silico* molecular docking outcomes. These molecules were also very mild cytotoxic towards membrane of the red blood cells. Hence, these molecules might be used as safe and promising drug candidates for urease-related ailments. 
